# The surgical outcome among children with congenital pseudoarthrosis of Tibia: Our 12 years of experience

**DOI:** 10.1371/journal.pone.0362359

**Published:** 2026-11-05

**Authors:** Tarun RajBhandari, Sabin Pokharel, Yam Prakash Gurung, Rajan Bhusal, Bibek Banskota, Ashok Kumar Banskota

**Affiliations:** Department of Orthopedics, Hospital and Rehabilitation Center for Disabled Children (HRDC), Janagal, Banepa, Nepal; Shuguang Hospital Affiliated to Shanghai University of Traditional Chinese Medicine, CHINA

## Abstract

**Background:**

Congenital Pseudoarthrosis of the Tibia (CPT) is a rare condition characterized by poorly perfused, invasive hamartomatous fibrous tissue that leads to fracture and pseudoarthrosis of the tibia. Management challenges include achieving union and preventing refractures. This study aims to evaluate surgical interventions and their outcomes in managing congenital pseudoarthrosis of the tibia in children.

**Materials and methods:**

This retrospective study reviewed 70 CPT cases treated between 2010 and 2022 at Nepal’s only specialized center for children with physical disabilities. It assessed demographics, surgical procedures, and outcomes, with techniques including periosteal grafting and the Masquelet method. Union was defined radiologically, and analyses were conducted using MS Excel and SPSS.

**Results:**

This study included 70 CPT patients; 40 male (57.1%) and 30 female (42.9%). The mean age at presentation was 3.84 years, with the first surgery at 5.38 years. Most cases were Boyd type 5 (71.43%) and Crawford type 4 (71.43%). Of 35 patients who underwent surgery, the union rate was 41.93% (13/31), with a refracture rate of 23.08% among unions. Periosteal grafting had a 50% union rate, while the Masquelet technique achieved 41.67%.

**Conclusions:**

This study highlights the challenges in managing Congenital Pseudoarthrosis of the Tibia (CPT), emphasizing the need for personalized surgical approaches based on patient age, limb length discrepancy, and classification. Effective patient counseling on risks and benefits is crucial for informed decisions.

## Introduction

Congenital Pseudoarthrosis of the Tibia (CPT) is a rare condition with an incidence of 1:140,000–1:250,000 live births [1]. It presents as pseudoarthrosis at birth or later as pathological fracture and develops anterolateral bowing of tibia, narrowing of medullary canal, or cyst [2]. The most common location is the junction of the middle and distal third of the tibia [2]. There is bone remodeling defect at the location [3]. Histologically fibrous hamartoma, abnormal periosteal thickening, and increased osteoclastic activity, creating mechanical interposition and defective vascularization at the CPT site [2]. The exact etiology is controversial but several theories have been put forward like genetic defect, fetal compression and amniotic bands damage, nutritional, hormonal or neurovascular defects [1]. These lead to pathological fracture and the development of pseudoarthrosis [2,4].

The tibia’s progressive anterolateral bowing leads to compensatory ankle valgus, fibular proximal migration, and limb shortening, hampering function [5–7]. The most challenging aspect of its management is achieving union and preventing refractures. When the patient presents before the fracture develops, bracing and protection of the limb is a part of non-operative treatment to delay or avoid the development of pseudoarthrosis [8]. The commonly used surgical techniques once the fracture has occurred or pseudoarthrosis has developed are intramedullary rod fixation, circular ring fixation, vascularized fibula grafting, Masquelet technique, combination and others [8]. However, due to persistent pain, recurrent fractures, or poor functional outcomes, amputation of the limb may be opted for [9]. Choi et al and Paley et al independently published the intentional cross union of the tibia and fibula for the treatment of CPT, which has a higher union rate and a lower refracture rate [10–12]. Different surgical strategies show a mean union rate of 72% (21–100%) and a mean refracture rate of 24% (0–68%) [12,13]. Multiple surgical techniques have been described for the management of CPT, each yielding variable outcomes. At our center, patients underwent different procedures over the study period, reflecting evolving surgical approaches informed by prior failures and emerging evidence, with the aim of improving patient outcomes.

This retrospective study aimed to find the outcomes of different approaches in managing Congenital Pseudoarthrosis of the Tibia (CPT) by examining the surgical procedures performed on patients at our center from January 2010 to December 2022. Specifically, the study evaluated the effectiveness of various surgical strategies in treating CPT, assessed the union rates achieved through these interventions, and calculated the incidence of refractures following surgical treatment.

## Materials and methods

This was a retrospective study of patients with CPT presenting to the outpatient clinic of the department of orthopedics of Hospital and Rehabilitation Center for Disabled Children (HRDC), Janagal, Banepa, Nepal, from January 2010 to December 2022. With the total sampling, all patients with CPT presenting to the center who were less than 18 years of age during the study period were included in the study. Patients with incomplete data in the medical directory and those without X-ray pictures were excluded from the study.

We accessed the hospital’s electronic database using the patient’s unique identification number. The patient’s data were retrieved from the hospital’s electronic record. The patient’s name, age, gender, age at first presentation, age at first surgery, involvement, method of management, and status of the union were looked for and recorded on the proforma. Radiological images of the CPT site were independently reviewed by at least two authors to classify the types of the lesion [12,14,15]. In case of discordant classification, a senior author gave the final decision. The status of the fracture site was assessed at the final follow-up, defined as the most recent hospital visit during which an X-ray was obtained. The bony union of the CPT site was defined radiologically when the fracture gap is bridged, the fracture line disappears, and at least three out of four cortices are also bridged [16]. The bony union was confirmed by two senior authors. The patient’s hospital records were reviewed to confirm the documentation of the clinical union, that was defined as the absence of pain at the fracture site on weight bearing, no tenderness at fracture site on palpation, and ability to bear weight [17]. Refracture was defined as when a fracture occurs at the original site or a new tibia site after a bony union had already occurred [18].

All the cases of CPT were managed by the same team of orthopedic surgeons and underwent surgery followed by close monitoring at regular three-month intervals. Except for those stabilized with Ilizarov rings, all patients were initially immobilized in a posterior slab until suture removal on postoperative day 14. After the removal of sutures, an above-knee cast was applied and changed at each follow up. Non-weight-bearing mobilization with crutches was advised for all cases. In patient where union was achieved, external bracing was given for protection, and internal fixation devices were retained to support the consolidation.

The periosteal grafting was done after excision of the pseudoarthrosis site and stabilizing it with an intramedullary rod, adding the periosteum graft harvested from the inner table of the iliac wing from underside of iliacus muscle wand supplementing it with Ilizarov external ring fixation as described by Thabet et al. [19] The inner cortex of the iliac wing was also harvested to be used for grafting. The Masquelet technique was used in 2 stages, as described in a study, stabilized with intramedullary non-telescopic rod supplemented with above-knee cast [20]. The rods were placed transarticular through the ankle joint and fibula was stabilized with an intramedullary wire. Ilizarov techniques were used to stabilize the tibia after resection of the pseudoarthrosis site, primary docking and cortico-cancellous bone autograft and no additional procedures [21]. Cross-union surgery was done as Paley et al. described and modified with a plate on the tibia instead of circular external fixation, but we did not use BMP due to its cost and unavailability [12]. This was supplemented with above-knee cast. The bisphosphonate infusion was given on two occasions, two weeks prior to and after the surgery, in order to decrease osteoclastic activity [1].

The data were subsequently entered into MS Excel 2010, where descriptive statistical analysis was performed. For the inferential analysis, SPSS was used. Central tendency was assessed using the mean and standard deviation (SD) or Median and Interquartile range(IQR), while descriptive analysis was conducted using frequency and percentage. For the continuous data, we tested for normality of the distribution. For inferential analysis we applied the chi-square test for the categorical variables and Mann Whitney U Test for the continuous not normally distributed data. A p-value of less than 0.05 was considered statistically significant.

### Ethical statement

The B&B Institutional Review Committee (B&BIRC-23–45), Nepal, granted ethical approval for the study. As this involved secondary data, and data were analyzed anonymously informed consent was not obtained, and strict measures were implemented to protect confidentiality. All patient data were encrypted and securely stored, ensuring privacy throughout the data collection, analysis, and interpretation. Access to sensitive information was restricted to authorized personnel only, and all steps were taken to prevent unauthorized access or disclosure.

## Results

The total data of 81 patients with CPT were retrieved from the hospital’s electronic database. Among them, 11 patients’ X-rays could not be retrieved, so they were excluded from the study. A total of 70 case details has been recorded. Among them, 8 cases from OPD and 2 cases seen at the camp were given elective surgery dates but were lost to follow-up Fig 1.

**Fig 1:**
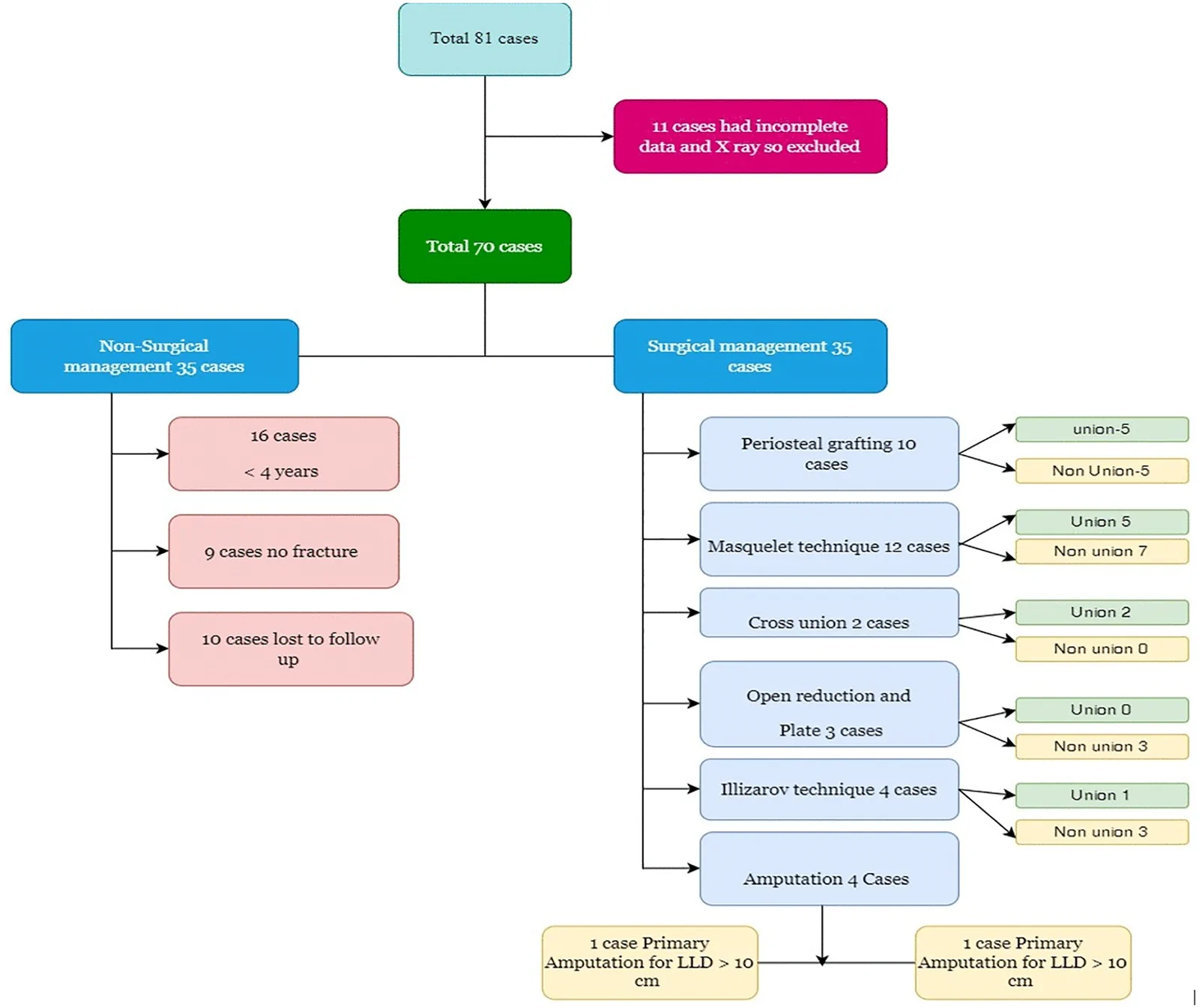
Flowchart showing the cases and the details of the procedure.

The patients of CPT were from all the provinces of Nepal, and 1 case was from India Table 1.

**Table 1:** General characteristics of the patients.

Particulars	Frequency	Percentage (%)
A. Gender (N = 70)		
*I. Female*	30	42.86%
*II. Male*	40	***57.14%***
B. Involvement (N = 70)		
*I. Bilateral*	3	4.29%
*II. Left*	34	48.57%
*III. Right*	33	47.14%
C. Address (N = 70)		
*I. Koshi Province*	11	15.71%
*II. Madhesh Province*	18	25.71%
*III. Bagmati Province*	17	24.29%
*IV. Gandaki Province*	5	7.14%
*V. Lumbini Province*	12	17.14%
*VI. Karnali Province*	1	1.43%
*VII. Sudurpaschim Province*	5	7.14%
*VIII. INDIA*	1	1.43%
D. Chief complaints (N = 70)		
*I. Deformity*	59	84.29%
*II. Inability to walk*	10	14.29%
*III. Swelling*	1	1.43%
E. Café au lait spots (N = 70)		
*I. No*	28	40.00%
*II. Yes*	42	60.00%
F. Weight-bearing status after the first presentation (N = 70)		
*I. Non-weight bearing*	14	20.00%
*II. Weight-bearing with crutches*	3	4.29%
*III. Weight-bearing with tibial guard*	53	75.71%
G. Tibia and fibula status (N = 70)		
*I. No bone fracture*	14	20.00%
*II. Both bone fracture*	40	57.15%
*III. Only tibia fracture*	11	15.71%
*IV. Only fibula fracture*	**5**	7.14%
H. Ankle valgus deformity/ distal fibula proximal migration (N = 70)		
*I. Yes*	23	32.86%
*II. No*	47	67.14%
I. Union achieved among cases undergoing surgical management for union (N = 31)		
*I. Yes*	13	41.94%
*II. No*	18	58.06%

The demographic characteristics of the patients are shown in Table 1. Among 35 cases of non-operative cohort, 16 cases are less than four years, so awaiting surgery, 9 cases have no fracture till final follow-up, and 10 cases were lost to follow-up. The mean age of presentation is 3.84 ± 3.14 years, and the age at first surgery is 5.38 **±** 2.46 years. Limb length discrepancy ranged from 0.5 cm to 14 cm, with a mean of 2.51 **±** 3.17 cm. The mean number of surgeries for a single patient is 2.84 **±** 1.39 Table 2.

**Table 2:** Descriptive data of age in years and Limb length discrepancy.

Particulars	Minimum	Maximum	Mean	SD
Age at first presentation in years	1	14	3.84	3.14
Age at first surgery in years	4	14	5.38	2.46
Limb length discrepancy (LLD) in centimeter(cm)	0.5	14	2.51	3.17
Number of surgeries	1	5	2.84	1.39

Most of the cases fell on Boyd type 5, 71.43% (n = 50/70), Crawford type 4, 71.43% (n = 50/70), and Paley 4A 30% (n = 21/70) Table 3.

**Table 3:** Classification of CPT according to Boyd [14], Crawford [15], and Paley's [12] method.

Classification	Boyd		Crawford		Paley	
	Types	Number (%)	Types	Number (%)	Types	Number (%)
	1	1(1.43%)	1	17(24.29%)	1	14(20.00%)
	2	13(18.57%)	2	2(2.86%)	2A	2(2.86%)
	3	1(1.43%)	3	1(1.43%)	2B	1(1.43%)
	4	5(7.14%)	4	50(71.43%)	3	9(12.86%)
	5	50(71.43%)			4A	21(30.00%)
	6	0(0%)			4B	12(17.14%)
					4C	11(15.71%)

Different surgeries were attempted on 35 patients for the management of CPT. The bar diagram below depicts the union rate within the mean follow-up period of 4.29 years Fig 2.

**Fig 2:**
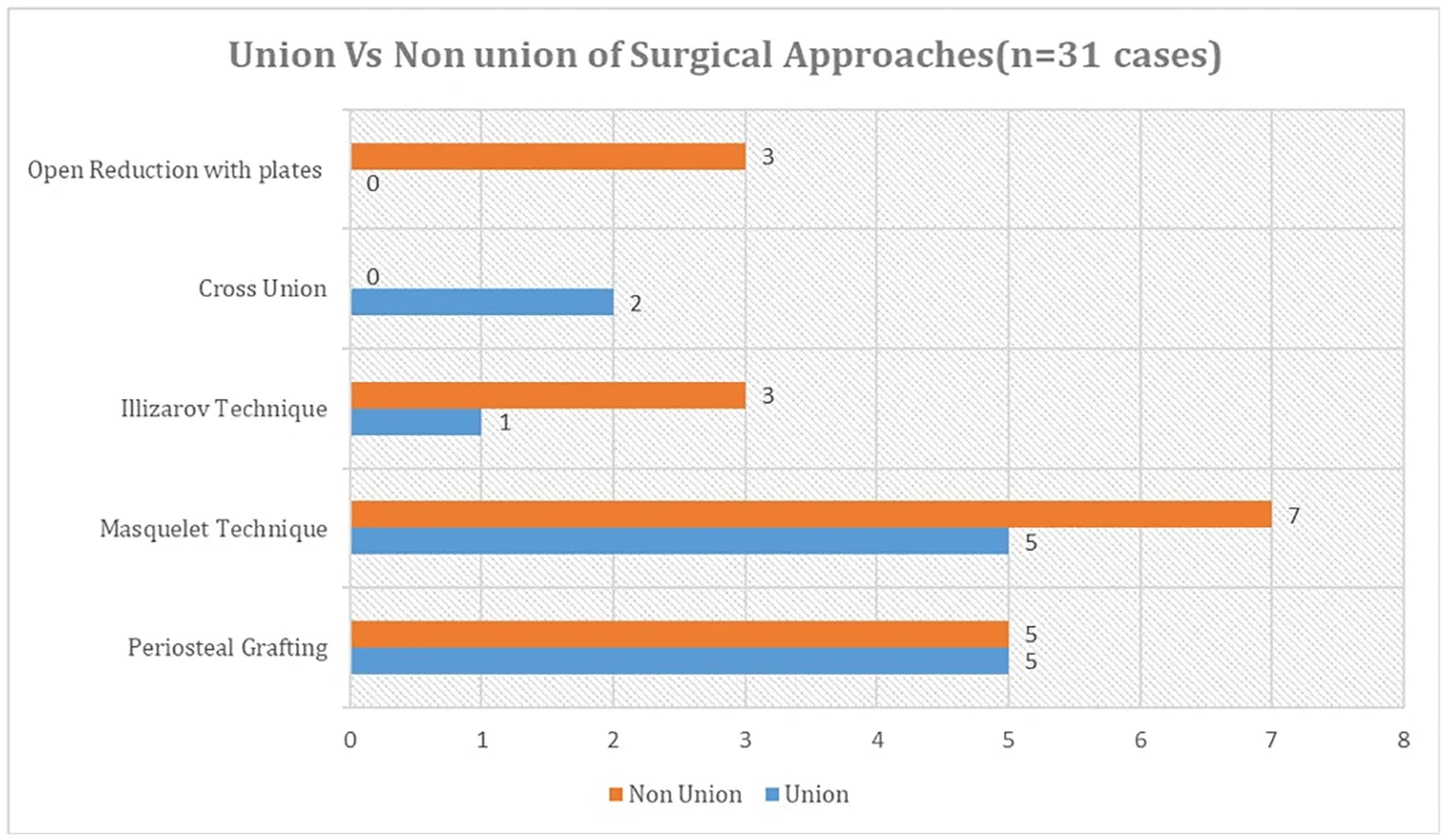
Bar diagram showing the union and non-union numbers of different surgical approaches for the CPT.

Thirty-one cases were included for calculating union or non-union rates, excluding the four amputations Fig 2. Therefore, the overall union rate was 41.93% (n = 13/31). The surgically managed cases had a median follow-up of 21 months (IQR 28), ranging from 1 to 72 months. There was one case with follow up less than 9 months, it was classified as non union for the analysis to prevent selection bias among the included surgical cases. Among 13 unions, 23.08% (n = 3/13) cases had refracture. Our study treated most cases using Periosteal grafting and the Masquelet technique. Periosteal grafting achieved a 50% (n = 5/10) union rate, and the Masquelet technique achieved a 41.67% (n = 5/12) union rate [1]. The Limb length discrepancy, age at first presentation, age at first surgery, presence of café au lait spots, and ankle proximal migration were not statistically significant to union rates Table 4.

**Table 4:** Union compared to different parameters.

Parameters		n	Median (IQR)	p-value
Age at first presentation	Union	13	3 (5)	0.352^a^
	Non-union	18	4 (4)	
Age at first surgery	Union	13	4 (2)	0.890 ^a^
	Non-union	18	4(2)	
Limb length discrepancy	Union	13	5 (6.5)	0.708^a^
	Non-union	18	5 (3.3)	
Café au lait spots	Union	13		0.253^b^
	Non-union	18		
Ankle proximal migration	Union	13		0.524^b^
	Non-union	18		

^a^ **Mann Whitney U test**

^b^ **Pearson’s Chi square test**

## Discussions

The total achieved union rate was 41.93% (n = 13/31), and the refracture rate was 23.08% (n = 3/13). This study was carried out at a referral center in the country, so the caseload is higher than that of other reported studies [2,4–8,22–24]. Most cases fell into the Boyd Type 5 or Crawford Type IV classification, similar to a study done in China but different from other studies [14,25,26]. The Union rate and the refracture rate were comparable to many reported studies [5,6,23,27]. More male patients had CPT, but side preference was nearly equal, similar to previous findings [6,18,23,28].

Secondary changes like LLD and ankle valgus need to be accounted for achieving satisfactory results. Fibula fracture contributes to proximal migration of the fibula and ankle valgus development. In our cases, ankle proximal migration did not alter the union rates (p = 0.524) though we rodded the fibula. CPT is one condition where growth of the tibia is inhibited, whereas the femur may overgrow as compensation [1]. Our cases had mean LLD of 2.51 (SD 3.17) ranging from 0.5 to 14 cm and it was not different in union and non-union groups (p = 0.708). LLD variability could be due to different ages of presentation.

Most of the amputations in our series were after multiple failed surgeries and underwent Below Knee Amputation (BKA). In a retrospective study of CPT cases treated with amputation, nearly 70% (n = 9/13) cases achieved the primary union of pseudoarthrosis after Boyd amputation, excision of CPT site, and stabilization with Intramedullary devices. However, 23.52% (n = 4/17) cases underwent primary BKA, and two more cases were converted to BKA after persistent non-union after Boyd amputation. However, 76% (n = 13/17) of cases required additional surgeries even after amputation [29]. So amputation is rarely recommended [5]. In our study, one case with an LLD greater than 10 cm underwent primary amputation. Additionally, three other cases required below-knee amputation (BKA) following multiple failed surgeries. In a prospective study by Meselhy MA, the induced membrane or Masquelet technique resulted in bony union in 100% (n = 20/20) cases. No refracture was seen until the mean follow-up of nearly five years [30]. Our results were likely lower because the other study used the Ilizarov apparatus as the external immobilizer, while we used an intramedullary rod and cast for stabilization. In most cases with concomitant fibular dysplasia in our series, the fibula was not stabilized, potentially leading to less rigid fixation at the tibial pseudoarthrosis site. The radiographic union time may be delayed in this technique, and secondary procedures for ankle or limb length discrepancy are still required [5].

In a study by Kocaoğlu et al on managing CPT using the periosteal grafting technique, 88.23% (15 out of 17) of cases achieved primary union, while 33.33% (5 out of 15) experienced refracture [31]. The lower union rate in our cases may be attributed to the failure to achieve union at the fibular pseudarthrosis, which could have led to instability at the tibial pseudarthrosis site.

In a multicenter study involving patients with CPT, the union rate for surgeries performed after 1995 was reported to be 69%, which is higher than the rate observed in our study. This discrepancy may be attributed to the use of vascularized grafts in the former study, which likely contributed to the increased union rate [23]. Vascularized fibular grafting is difficult for resource-constrained settings like ours. In the study done in 255 cases of CPT of Crawford type IV operated between 2013 and 2020 done by Li Z Yu H, 92.5% (n = 236/255) of cases achieved primary bone union, and 13.3%(n = 34/255) had refracture [28]. They used a combined surgery approach with cross union, and in most cases, they used external and internal fixation. Similarly, a study of 119 cases from 6 centers in different countries shows 86% (n = 102/119) achieved union after index operations, but 40 cases had refractured [18]. In a study by Horn J, 66% (n = 10/15) cases achieved union using Ilizarov fixation [6]. Data from different hospitals in Germany in a study by Laufer A have shown that 69% (n = 18/26) cases had a primary union, and in four cases, 22% (n = 4/18) had refractures [27].

In 2002, Johnston et al. reported the use of cross-union for the CPT [32]. Later, Choi et al. converged the two fibula bone ends towards the two tibia bone ends, used the bone graft from the ilium, and created a bridging bone mass in CPT, termed “4-in-1 osteosynthesis” [10].

Paley independently reported cross-union technique while keeping the tibia and fibula rodded apart by their normal interosseous distance, with a bony graft bridge achieved. He combined pharmacological treatment with Zoledronic acid (ZA) and BMP-2 to prevent the resorption of bone grafts and accelerate osteogenic response. Unlike other reported studies, he resected a minimal of the tibia to achieve mechanical alignment, as the tibial bone is viable and not the cause of CPT non-union [33]. For the cross-union surgeries, we did not have BMP available with us, but we tried to adhere to the steps outlined. Authors have reported unions in cases managed by them at the places where BMP is unavailable [34]. Therefore, the use of BMP may not always be necessary.

The decancellousization of the ilium creates a hollow but has no reported effect on hip development, the center-edge angle (CEA), and the acetabular index (AI) as compared to the unharvested side [34]. Intentional cross union achieved between the tibia and fibula gives mechanical stability, creates a suitable biological environment for union, and a thicker bony bridge decreases the chances of refracture [35]. Another pharmacotherapeutic adjunctive used in these cases was recombinant human BMP7, which yielded unsatisfactory results [34]. Choi et al. achieved a bony bridge in all eight cases without using BMP [10]. So, BMP accelerates the osteogenic response, but other factors like mechanical alignment, stability, and fibula status also affect the union rates [33]. The use of umbilical cord mesenchymal stem cells (UC-MSCs) and secretome after resection of hamartoma and fixing with plate and screws in CPT has yielded union in 5 out of 6 cases [36]. Hence, the exploration of the use of more adjunctive therapeutic procedures in CPT is required.

A retrospective study of 39 cases using cross-union protocol with internal fixation, bone grafting, zoledronic acid infusion, and bone morphogenic protein 2 (BMP2) insertion showed the union rate approaching 100%(n = 39/39) with no refractures till seven years of post-operative follow-up [37]. In a recent systematic review of 1227 cases of CPT, all cases of CPT undergoing cross-union surgery united within 4.5 months with a low refracture rate of 22.5%. The probability of successful cross-union surgery for CPT approached 77.5%. Using intramedullary rods achieved a union rate of 67.7% within 12.6 months and a success probability of 35.2% [38].

Our study utilized internal and external fixation methods, periosteal grafting, and the Masquelet technique with an intramedullary rod. We achieved union in 41.93% (13 out of 31) of the cases undergoing surgery. This rate is lower than that reported in other studies. The discrepancy may be due to the ongoing follow-up of many cases and the high cost and unavailability of BMP in our setting. However, a systematic review and meta-analysis have refuted the use of BMP for primary union [7]. The same study showed a 75% (n = 300/401) union rate within 7.2 months and a 35% (n = 141/401) refracture rate.

Cross-union surgeries and combined surgeries using the internal and aided by external fixation methods have shown a reasonable union rate with low refracture, so a prospective study for these surgeries done by the same surgeon on CPT cases will further confirm the findings.

### Limitations and opportunities for further study

The study on Congenital Pseudoarthrosis of the Tibia (CPT) has notable limitations, including its retrospective design, which restricts control over confounding variables and potential biases. The sample size of 35 cases may lack the power to detect significant differences among surgical techniques, and excluding patients with incomplete data could introduce selection bias. Additionally, the follow-up period may be insufficient for assessing long-term outcomes. We intentionally classified one case with follow-up of less than 9 months as non-union for analysis purposes. By selectively omitting a surgically managed case, we may have introduced selection bias; therefore, we did not exclude the case from the analysis. However, this may have lowered the reported union rate. Given that this represents a single case, the impact on the union rate may be minimal, and the principal conclusion of the study remains unchanged. Nevertheless, studies with longer follow-up could improve the accuracy of the union rates.

While variability in surgical approaches and the single-center scope limit the generalizability of the findings, the small sample size further limits interpretation. The clear indications of the selection of different surgical techniques were not consistently documented in the hospital database; hence we were unable to report it. Due to the small sample size, we were not able to carry out the multivariable analysis.

Despite these limitations, several opportunities for further research exist. Prospective studies with standardized protocols could improve data reliability, and multicenter trials would enhance sample diversity. Longitudinal studies could provide insights into the sustainability of surgical outcomes over time. Investigating adjunctive therapies, such as Platelet Rich Plasma, PRP, or stem cell treatments, may yield improved union rates. Incorporating qualitative assessments of patient-reported outcomes would deepen understanding of the impact of CPT treatment on quality of life. Addressing these limitations and pursuing these research avenues could significantly advance the management of CPT and improve patient outcomes.

## Conclusions

This study highlights the complex challenges associated with the surgical management of Congenital Pseudoarthrosis of the Tibia (CPT), revealing a union rate of 41.93% and a refracture rate of 23.08%. While age and classification types were noted as potential factors, their direct impact on surgical strategy was not established in this study, warranting further investigation. Future research should focus on defining evidence-based surgical approaches tailored to specific patient profiles. Additionally, prospective studies with standardized surgical techniques and longer follow-up periods are needed to validate these findings and explore new treatment strategies.

## Supporting information

S1 Data For Plos1. (XLSX)
