# Supplementary material for: The surgical outcome among children with congenital pseudoarthrosis of Tibia: Our 12 years of experience
Source: PLoS One. 2026 Nov 5;21(11):e0362359. doi: 10.1371/journal.pone.0362359 (PMC13739177; doi:10.1371/journal.pone.0362359)
Supplement: Supplementary file 1 [file pone.0362359.s002.pdf]

Dear Editorial team and the Reviewers,
We, the authors, are grateful to the editorial team and the reviewers for their careful evaluation of our manuscript and for providing valuable suggestions. Their insightful comments have enabled us to refine and improve the manuscript into a stronger version.

The point wise replies are given below:

Comments	Authors reply
Editor's comments	
1. Please ensure that your manuscript meets PLOS ONE's style requirements, including those for file naming. The PLOS ONE style templates can be found at https://journals.plos.org/plosone/s/file?id=wjVg/PLOSOne_formatting_sample_main_body.pdf and https://journals.plos.org/plosone/s/file?id=ba62/PLOSOne_formatting_sample_title_authors_affiliations.pdf	Dear Editor,<br>We have revised and corrected the manuscript according to the journal requirement. We revised according to the template.
2. Your ethics statement should only appear in the Methods section of your manuscript. If your ethics statement is written in any section besides the Methods, please move it to the Methods section and delete it from any other section. Please ensure that your ethics statement is included in your manuscript, as the ethics statement entered into the online submission form will not be published alongside your manuscript.	We have revised and moved the ethical statement under the methods section.

3. . Please remove your figures from within your manuscript file, leaving only the individual TIFF/EPS image files, uploaded separately. These will be automatically included in the reviewers' PDF.	We have removed the figures and changed the format to tiff, which will be uploaded as a separate file.
4. . We note that there is identifying data in the Supporting Information file "Supple.xlsx". Due to the inclusion of these potentially identifying data, we have removed this file from your file inventory. Prior to sharing human research participant data, authors should consult with an ethics committee to ensure data are shared in accordance with participant consent and all applicable local laws. Data sharing should never compromise participant privacy. It is therefore not appropriate to publicly share personally identifiable data on human research participants. The following are examples of data that should not be shared:<br><br>-Name, initials, physical address<br>-Ages more specific than whole numbers<br>-Internet protocol (IP) address<br>-Specific dates (birth dates, death dates, examination dates, etc.)<br>-Contact information such as phone number or email address<br>-Location data<br>-ID numbers that seem specific (long numbers, include initials, titled "Hospital ID") rather than random (small numbers in numerical order)<br><br>Data that are not directly identifying may also be inappropriate to share, as in combination they can become identifying. For example, data collected from a small group of participants, vulnerable populations, or private groups	Dear editors,<br>we apologize for the inclusion of age and location in the data. We have revised and removed such identifying information.

should not be shared if they involve indirect identifiers (such as sex, ethnicity, location, etc.) that may risk the identification of study participants.<br><br>Additional guidance on preparing raw data for publication can be found in our Data Policy (https://journals.plos.org/plosone/s/data-availability#loc-human-research-participant-data-and-other-sensitive-data) and in the following article: http://www.bmj.com/content/340/bmj.c181.long.<br><br>Please remove or anonymize all personal information "Age and Location", ensure that the data shared are in accordance with participant consent, and re-upload a fully anonymized data set. Please note that spreadsheet columns with personal information must be removed and not hidden as all hidden columns will appear in the published file.	
5.If the reviewer comments include a recommendation to cite specific previously published works, please review and evaluate these publications to determine whether they are relevant and should be cited. There is no requirement to cite these works unless the editor has indicated otherwise.	Dear editors,<br>We have changed the manuscript according to the suggestions made by the reviewers.

Reviewer 1	
Interesting topic , as this study contributes useful clinical data on a rare orthopedic condition. That was taken into consideration while reviewing this submission.<br><br>Here are my comments:<br><br><br>The Introduction is too brief and lacks sufficient background. While the authors introduce congenital pseudoarthrosis of the tibia and state the study objectives, the literature review is limited. A more comprehensive overview of current surgical management strategies, existing controversies, knowledge gaps, and the rationale for conducting this study would better establish its scientific significance.	Dear reviewer,<br>We have expanded on the introduction section. We have added literature review as well.
The Methods section requires substantial clarification. It is not immediately clear how the study was conducted from start to finish. The methodology should be reorganized to provide a clearer description of patient identification, eligibility criteria, data collection procedures, follow-up assessment, outcome definitions, and the overall study workflow. The current presentation makes it difficult for readers to fully understand and reproduce the study.	We have revised the methods section. We have tried to make it clearer. The criteria for identification, data collection methodology, follow up assessment and the overall workflow has been defined according to the suggestions made.
the criteria for selecting each surgical technique are insufficiently described. It remains unclear why particular patients	This is a retrospective data and we have collected the data regarding the surgery done in our cases. The use of different surgical techniques reflects evolving surgical approaches

underwent periosteal grafting, Masquelet technique, Ilizarov fixation, cross-union, or amputation. Without clearly defined treatment indications, interpretation and comparison of surgical outcomes are limited.	informed by prior failures and emerging evidence, with the aim of improving patient outcomes at the study center and has been added to the manuscript. However, clear indications for the selection of each technique were not consistently documented in the database, and therefore could not be reported. This has been added to the limitation section.
The statistical analysis is relatively basic. Although appropriate statistical tests were performed, the analysis is limited to univariate comparisons. Given the availability of multiple clinical variables, the authors should justify why multivariable analysis was not performed or acknowledge this limitation more explicitly.	Due to small sample size we could not perform multivariable analysis. This has now been listed under the limitation section.
The discussion occasionally overinterprets the findings. Since this is a retrospective single-center study with relatively small numbers in each surgical subgroup, statements regarding the effectiveness of individual techniques should be interpreted more cautiously.	We have been more cautious in interpretation of our findings in the discussion and changed the statements accordingly.
The follow-up reporting could be improved. In addition to the mean follow-up duration, reporting the range or median follow-up would provide a clearer understanding of outcome assessment and the evaluation of refracture	We have added the follow up data.
Careful proofreading would improve readability and presentation.	We have done careful proofreading and checked for grammatical errors.
Reviewer 2	
The abstract states that the study included 40 male and 30 female patients, but Table 1 lists 45 male (64.29%) and 25 female (35.71%) patients. This is a major internal contradiction that must be resolved.	Dear reviewer,<br>We apologize for the typo. We have corrected it.<br>The contradictions in the table and the abstract has been resolved. The data of the table is the correct one.

Classifying 35 patients under "non-surgical treatment/management" is conceptually problematic because this cohort includes 16 patients who are simply awaiting surgery (under 4 years old), 9 patients with no fracture, and 10 patients who were lost to follow-up These patients did not receive non-operative "treatment" and should be classified as a non-operative cohort or excluded/tracked separately.<br>Table 1 Percentage Error: For the 31 cases evaluated for union, Table 1 states that 13 patients achieved union and represents this as 41.39%.<br>Mathematically, 13 out of 31 is actually 41.94% (which is correctly listed elsewhere in your abstract and results	
Title Typo: The title contains a clear typographical error: "Our 12 1 Years of Experience"<br>. It should be corrected to "12 Years" to reflect the study period from 2010 to 2022	Dear reviewer,<br>we apologize for the typing error. But the file downloaded from the system failed to show such typo. However, we have made sure that it does not occur.
the discussion claims that periosteal grafting and the Masquelet technique demonstrated a "higher union rate than other methods", yet your results show that Cross-union actually had a 100% union rate (2/2 cases)<br>, and the overall union rates for grafting (50%) and Masquelet (41.67%) are lower than the international literature. The text needs to be rephrased to be more objective and address these rates in the context of resource limitations (such as the lack of BMP	We have rephrased the statement to correct our over statement.
When analyzing categorical variables—such as Café au lait spots (presence/absence), Ankle proximal migration (yes/no), and Side of involvement (left/right/bilateral)—stratified by union status, the expected frequency in many cells of the	We carried out the analysis again. But the contingency table has all the cells with more than expected counts(>5 per cells). Hence, we continued with our chi square tes.

contingency tables will inevitably be less than 5<br>Re-analyze categorical variables in Table 4 using Fisher's Exact Test instead of the Chi-square test	
Re-analyze continuous variables (particularly LLD) using the non-parametric Mann-Whitney U test to account for non-normality and small subgroup sizes	We checked the data for normality and carried out the Mann-Whitney U test which has been explicitly listed in the table 4.
Completely restructure Table 4 to include:<br>Subgroup counts and percentages for all categorical variables<br>.<br>Subgroup standard deviations (SD) for continuous variables<br>.<br>Explicit test statistics (e.g., U statistic, t-statistic, df, and 95% Confidence Intervals for differences).	We have reanlayzed the data and presented the result accordingly. We have included the Median and IQR for the non normal continuous data. For the categorical data we have used the pearson's chi square test.
Expand the Limitations section to discuss how the small sample size (N=31) prevented multivariable regression, leaving the significant correlation between LLD and union unadjusted for potential clinical confounders	We have added to the limitation section about the lack of multivariate analysis.
**Reviewer 3**<br><br>The manuscript addresses an important and challenging pediatric orthopedic condition and presents a relatively large single-center experience with CPT over a 12-year period. However, substantial revision is required before the findings can be adequately interpreted.	
INTRODUCTION<br>• Introduction is too brief and would benefit from further elaboration.<br>• Expand on the clinical significance and	Dear reviewer,<br>• We have expanded on the introduction section. The natural history, presentation and the surgical options have been discussed explicitly now.

natural history of CPT.<br>• Provide more background on the challenges of achieving and maintaining union.<br>• Briefly summarize the main surgical options currently used for CPT.<br>• Discuss the wide variation in reported union and refracture rates.<br>• Highlight the limitations or gaps in the existing literature.<br>• The sentence "The pathology involves poorly perfused invasive hamartomatous fibrous tissue in the surrounding periosteum having increased osteoclasis, low osteogenesis and bone morphogenetic protein" must be revised.<br>• The inclusion of bone morphogenetic protein in this sentence is unclear and appears incomplete.<br>• Please clarify the specific role or alteration of BMP in CPT pathology (e.g., reduced expression/activity), rather than simply listing it alongside increased osteoclasis and reduced osteogenesis.<br>• If the authors are specifically referring to BMP-2, this should be clearly stated and supported by the cited reference.	• The sentence "The pathology involves poorly perfused invasive hamartomatous fibrous tissue in the surrounding periosteum having increased osteoclasis, low osteogenesis and bone morphogenetic protein" has been revised.<br>• The statement regarding the BMP had been revised to make it clearer.
METHOD<br>• Consider dividing the Methods into subsections such as Study design and setting; Patient selection; Surgical indications and techniques; Postoperative management and follow-up; Outcome definitions; Ethical approval; and Statistical analysis	We have added substantially to the methods section. Since all the required data could not be located in the database ,appropriately subsections could not be managed. However, ethical statement has been added to the methods section.
The Methods section requires substantial elaboration to allow reproducibility and proper interpretation of the surgical outcomes.<br>• The process of patient identification and selection should be described more clearly. Since this is a retrospective study, the authors should explain how eligible CPT cases were identified from the	• We have elaborated on the methods section to make it clearer to the reader and allow for the reproducibility.<br>• The patients selection criteria has been listed. The overall flow diagram of the patients has been shown in the figure 1.<br>• The total sampling method was applied which has been added to the methods section.<br>• The flowchart is given in the figure 1

hospital database, how many records were screened, and the reasons for exclusion.<br>• Please clarify whether all consecutive eligible patients treated during the study period were included, or whether any sampling method was used.<br>• A clear patient selection flowchart should be provided, showing the number of patients screened, excluded, managed non-operatively, operated on, lost to follow-up, and included in the final union/refracture analysis.<br>• The indications for surgery are not adequately described. Please specify how the decision for operative treatment was made, including age, fracture status, deformity severity, previous treatment, limb-length discrepancy, or other relevant factors.<br>• The criteria used to select each surgical technique are unclear. The manuscript describes periosteal grafting, Masquelet technique, Ilizarov fixation, and cross-union, but does not explain why one procedure was chosen over another for a particular patient.<br>• The surgical protocols need more detail, including extent of pseudoarthrosis resection, fixation method, bone graft source, management of the fibula, use of external fixation/casting, and any adjunctive treatment.<br>• Please indicate whether procedures were performed by the same surgeon or multiple surgeons, and whether the surgical technique changed over the 12-year study period.<br>• The postoperative protocol is insufficiently described.	• The use of different surgical techniques reflects evolving surgical approaches informed by prior failures and emerging evidence, with the aim of improving patient outcomes at the study center and has been added to the manuscript. However, clear indications for the selection of each technique were not consistently documented in the database, and therefore could not be reported. This has been added to the limitation section.<br>• The surgical protocols have been elaborated.<br>• The cases were managed by the same orthopedic team involving multiple surgeons. However the surgical techniques for each procedures followed was the same. This has been explained and added to the manuscript.<br>• The post operative protocol followed has been listed in the manuscript.
The authors should clarify the minimum and mean follow-up duration required for assessment of union and refracture.	We have added the median follow-up duration.

the definition of union is provided, but it should be clarified who assessed the radiographs, whether assessment was performed independently, and how disagreement was resolved.	The bony union was confirmed by two senior authors. The patient's hospital records were reviewed to confirm the documentation of the clinical union, that was defined as the absence of pain at the fracture site on weight bearing, no tenderness at fracture site on palpation, and ability to bear weight<br>This has been added to the manuscript
The ethical approval statement should be incorporated clearly within the Methods section, rather than appearing only later in the manuscript.	We have moved the ethical statement to the methods section.

Corresponding author

Sabinpokh100@gmail.com
