# Supplementary material for: The surgical outcome among children with congenital pseudoarthrosis of Tibia: Our 12 years of experience
Source: PLoS One. 2026 Nov 5;21(11):e0362359. doi: 10.1371/journal.pone.0362359 (PMC13739177; doi:10.1371/journal.pone.0362359)
Supplement: Supplementary file 2 [file pone.0362359.s003.pdf]

Dear Editorial team and the Reviewers,
We, the authors, are grateful to the editorial team and the reviewers for their careful evaluation of our revised manuscript and for accepting most of the revisons. However, below are the point to point response to the additional comments.

The point wise replies are given below:

Comments	Authors reply
If the authors have adequately addressed your comments raised in a previous round of review and you feel that this manuscript is now acceptable for publication, you may indicate that here to bypass the “Comments to the Author” section, enter your conflict of interest statement in the “Confidential to Editor” section, and submit your "Accept" recommendation.<br><br>Reviewer #1: All comments have been addressed<br><br>Reviewer #2: All comments have been addressed	We are grateful to the reviewers for accepting the revisions.
Is the manuscript technically sound, and do the data support the conclusions?<br><br>The manuscript must describe a technically sound piece of scientific research with data that supports the conclusions. Experiments must have been conducted rigorously, with appropriate controls, replication, and sample sizes. The conclusions must be drawn appropriately based on the data presented.<br><br>Reviewer #1: Partly<br><br>Reviewer #2: Yes	We are grateful to the reviewers for accepting the revisions.

Has the statistical analysis been performed appropriately and rigorously?<br><br>Reviewer #1: Yes<br><br>Reviewer #2: Yes	We are grateful to the reviewers for accepting the revisions.
. Have the authors made all data underlying the findings in their manuscript fully available?<br><br>The PLOS Data policy requires authors to make all data underlying the findings described in their manuscript fully available without restriction, with rare exception (please refer to the Data Availability Statement in the manuscript PDF file). The data should be provided as part of the manuscript or its supporting information, or deposited to a public repository. For example, in addition to summary statistics, the data points behind means, medians and variance measures should be available. If there are restrictions on publicly sharing data—e.g. participant privacy or use of data from a third party—those must be specified.<br>Reviewer #1: Yes<br>Reviewer #2: Yes	We are grateful to the reviewers for accepting the revisions.
. Is the manuscript presented in an intelligible fashion and written in standard English?<br><br>PLOS ONE does not copyedit accepted manuscripts, so the language in submitted articles must be clear, correct, and unambiguous. Any typographical or grammatical errors should be corrected at revision, so please note any specific errors here.	We are grateful to the reviewers for accepting the revisions..

Reviewer #1: Yes<br>Reviewer #2: Yes	
Review Comments to the Author<br><br>Please use the space provided to explain your answers to the questions above. You may also include additional comments for the author, including concerns about dual publication, research ethics, or publication ethics. (Please upload your review as an attachment if it exceeds 20,000 characters)	
Reviewer #1: Two issues remain that should be addressed before publication.<br><br>1. Follow-up duration and classification of non-union: The surgically managed patients had a median follow-up of 21 months (IQR 28), but the reported range is very wide, from 1 to 72 months. A follow-up of only one month would generally be insufficient to establish a definitive surgical outcome, particularly non-union or subsequent refracture. This issue is particularly important because the Discussion states that union has not yet been confirmed in some recently operated cases that remain under follow-up. The authors should clarify how many patients had relatively short follow-up and whether such patients were classified as non-union. If patients with insufficient follow-up were included within the non-union group, the denominator and reported overall union rate should be reconsidered or, at minimum, clearly justified.	Dear reviewer, thank you highlighting this important point. We have one case with short follow up. But we could not exclude the patient for analysis, as it would introduce selection bias among the surgically managed cases. However, the effect it being classified as the non union might have slightly lowered the union rates and the refracture rates. This point has been added to the result section and acknowledged in the limitation section as well.
Interpretation of outcomes between surgical techniques: The authors appropriately acknowledge that clear indications for selecting the different	Dear reviewer, thank you for a very valid point. We were very cautious in interpretation of the union rates among different procedures. We tried to avoid the comparison between

surgical techniques were not consistently documented. This is an important limitation because treatment allocation was therefore neither standardized nor controlled. Consequently, the reported union rates for periosteal grafting, Masquelet technique, and the other procedures should remain descriptive and should not imply comparative effectiveness between techniques. This limitation should remain clear throughout the Results, Discussion and Conclusions.	procedures. However, we have used descriptives in each surgical strategies.
Reviewer #2: author has addressed all the required comments and also improve the writing as requested from my previous comments	We are grateful to the reviewers for accepting the revisions.

Corresponding author

Sabinpokh100@gmail.com
