# Supplementary material for: The surgical outcome among children with congenital pseudoarthrosis of Tibia: Our 12 years of experience
Source: PLoS One. 2026 Nov 5;21(11):e0362359. doi: 10.1371/journal.pone.0362359 (PMC13739177; doi:10.1371/journal.pone.0362359)
Supplement: Peer review history

# Peer review: Decision Letter 0

**DOI:** 10.1371/journal.pone.0362359.r001

**Authors:** Xiaoen Wei

13 Aug 2026

Dear Dr. Pokharel,

Thank you for submitting your manuscript to PLOS ONE. After careful consideration, we feel that it has merit but does not fully meet PLOS ONE’s publication criteria as it currently stands. Therefore, we invite you to submit a revised version of the manuscript that addresses the points raised during the review process.

Please submit your revised manuscript by Oct 12 2026 11:59PM. If you will need more time than this to complete your revisions, please reply to this message or contact the journal office at plosone@plos.org. When you're ready to submit your revision, log on to https://www.editorialmanager.com/pone/ and select the 'Submissions Needing Revision' folder to locate your manuscript file.

- A letter that responds to each point raised by the academic editor and reviewer(s). You should upload this letter as a separate file labeled 'Response to Reviewers'.
- A marked-up copy of your manuscript that highlights changes made to the original version. You should upload this as a separate file labeled 'Revised Manuscript with Track Changes'.
- An unmarked version of your revised paper without tracked changes. You should upload this as a separate file labeled 'Manuscript'.

If you would like to make changes to your financial disclosure, please include your updated statement in your cover letter. Guidelines for resubmitting your figure files are available below the reviewer comments at the end of this letter.

If applicable, we recommend that you deposit your laboratory protocols in protocols.io to enhance the reproducibility of your results. Protocols.io assigns your protocol its own identifier (DOI) so that it can be cited independently in the future. For instructions see: https://journals.plos.org/plosone/s/submission-guidelines#loc-laboratory-protocols. Additionally, PLOS ONE offers an option for publishing peer-reviewed Lab Protocol articles, which describe protocols hosted on protocols.io. Read more information on sharing protocols at https://plos.org/protocols?utm_medium=editorial-email&utm_source=authorletters&utm_campaign=protocols.

As the corresponding author, your ORCID iD is verified in the submission system and will appear in the published article. PLOS supports the use of ORCID, and we encourage all coauthors to register for an ORCID iD and use it as well. Please encourage your coauthors to verify their ORCID iD within the submission system before final acceptance, as unverified ORCID iDs will not appear in the published article. *Only*  the individual author can complete the verification step; PLOS staff *cannot*  verify ORCID iDs on behalf of authors.

We look forward to receiving your revised manuscript.

Kind regards,

Xiaoen Wei

Academic Editor

PLOS One

Journal Requirements:

When submitting your revision, we need you to address these additional requirements.

1. Please ensure that your manuscript meets PLOS ONE's style requirements, including those for file naming. The PLOS ONE style templates can be found at

https://journals.plos.org/plosone/s/file?id=wjVg/PLOSOne_formatting_sample_main_body.pdf and

https://journals.plos.org/plosone/s/file?id=ba62/PLOSOne_formatting_sample_title_authors_affiliations.pdf

2. Your ethics statement should only appear in the Methods section of your manuscript. If your ethics statement is written in any section besides the Methods, please move it to the Methods section and delete it from any other section. Please ensure that your ethics statement is included in your manuscript, as the ethics statement entered into the online submission form will not be published alongside your manuscript.

3. Please remove your figures from within your manuscript file, leaving only the individual TIFF/EPS image files, uploaded separately. These will be automatically included in the reviewers’ PDF.

4. We note that there is identifying data in the Supporting Information file “Supple.xlsx”. Due to the inclusion of these potentially identifying data, we have removed this file from your file inventory. Prior to sharing human research participant data, authors should consult with an ethics committee to ensure data are shared in accordance with participant consent and all applicable local laws.

Data sharing should never compromise participant privacy. It is therefore not appropriate to publicly share personally identifiable data on human research participants. The following are examples of data that should not be shared:

-Name, initials, physical address

-Ages more specific than whole numbers

-Internet protocol (IP) address

-Specific dates (birth dates, death dates, examination dates, etc.)

-Contact information such as phone number or email address

-Location data

-ID numbers that seem specific (long numbers, include initials, titled “Hospital ID”) rather than random (small numbers in numerical order)

Data that are not directly identifying may also be inappropriate to share, as in combination they can become identifying. For example, data collected from a small group of participants, vulnerable populations, or private groups should not be shared if they involve indirect identifiers (such as sex, ethnicity, location, etc.) that may risk the identification of study participants.

Additional guidance on preparing raw data for publication can be found in our Data Policy (https://journals.plos.org/plosone/s/data-availability#loc-human-research-participant-data-and-other-sensitive-data) and in the following article: http://www.bmj.com/content/340/bmj.c181.long.

Please remove or anonymize all personal information “Age and Location”, ensure that the data shared are in accordance with participant consent, and re-upload a fully anonymized data set. Please note that spreadsheet columns with personal information must be removed and not hidden as all hidden columns will appear in the published file.

5. If the reviewer comments include a recommendation to cite specific previously published works, please review and evaluate these publications to determine whether they are relevant and should be cited. There is no requirement to cite these works unless the editor has indicated otherwise.

[Note: HTML markup is below. Please do not edit.]

Reviewers' comments:

Reviewer's Responses to Questions

**Comments to the Author**

1. Is the manuscript technically sound, and do the data support the conclusions?

Reviewer #1: Yes

Reviewer #2: Partly

Reviewer #3: Yes

**********

2. Has the statistical analysis been performed appropriately and rigorously?

Reviewer #1: N/A

Reviewer #2: Yes

Reviewer #3: No

**********

3. Have the authors made all data underlying the findings in their manuscript fully available?

The PLOS Data policy

Reviewer #1: Yes

Reviewer #2: Yes

Reviewer #3: Yes

**********

4. Is the manuscript presented in an intelligible fashion and written in standard English?

Reviewer #1: Yes

Reviewer #2: No

Reviewer #3: Yes

**********

Reviewer #1: Interesting topic , as this study contributes useful clinical data on a rare orthopedic condition. That was taken into consideration while reviewing this submission.

Here are my comments:

The Introduction is too brief and lacks sufficient background. While the authors introduce congenital pseudoarthrosis of the tibia and state the study objectives, the literature review is limited. A more comprehensive overview of current surgical management strategies, existing controversies, knowledge gaps, and the rationale for conducting this study would better establish its scientific significance.

The Methods section requires substantial clarification. It is not immediately clear how the study was conducted from start to finish. The methodology should be reorganized to provide a clearer description of patient identification, eligibility criteria, data collection procedures, follow-up assessment, outcome definitions, and the overall study workflow. The current presentation makes it difficult for readers to fully understand and reproduce the study.

The criteria for selecting each surgical technique are insufficiently described. It remains unclear why particular patients underwent periosteal grafting, Masquelet technique, Ilizarov fixation, cross-union, or amputation. Without clearly defined treatment indications, interpretation and comparison of surgical outcomes are limited.

The statistical analysis is relatively basic. Although appropriate statistical tests were performed, the analysis is limited to univariate comparisons. Given the availability of multiple clinical variables, the authors should justify why multivariable analysis was not performed or acknowledge this limitation more explicitly.

The discussion occasionally overinterprets the findings. Since this is a retrospective single-center study with relatively small numbers in each surgical subgroup, statements regarding the effectiveness of individual techniques should be interpreted more cautiously.

The follow-up reporting could be improved. In addition to the mean follow-up duration, reporting the range or median follow-up would provide a clearer understanding of outcome assessment and the evaluation of refracture.

Careful proofreading would improve readability and presentation.

Reviewer #2: The abstract states that the study included 40 male and 30 female patients, but Table 1 lists 45 male (64.29%) and 25 female (35.71%) patients. This is a major internal contradiction that must be resolved.

Classifying 35 patients under "non-surgical treatment/management" is conceptually problematic because this cohort includes 16 patients who are simply awaiting surgery (under 4 years old), 9 patients with no fracture, and 10 patients who were lost to follow-up

These patients did not receive non-operative "treatment" and should be classified as a non-operative cohort or excluded/tracked separately.

Table 1 Percentage Error: For the 31 cases evaluated for union, Table 1 states that 13 patients achieved union and represents this as 41.39%. Mathematically, 13 out of 31 is actually 41.94% (which is correctly listed elsewhere in your abstract and results

Title Typo: The title contains a clear typographical error: "Our 12 1 Years of Experience".

It should be corrected to "12 Years" to reflect the study period from 2010 to 2022

The discussion claims that periosteal grafting and the Masquelet technique demonstrated a "higher union rate than other methods", yet your results show that Cross-union actually had a 100% union rate (2/2 cases)

, and the overall union rates for grafting (50%) and Masquelet (41.67%)

are lower than the international literature. The text needs to be rephrased to be more objective and address these rates in the context of resource limitations (such as the lack of BMP

When analyzing categorical variables—such as Café au lait spots (presence/absence), Ankle proximal migration (yes/no), and Side of involvement (left/right/bilateral)—stratified by union status, the expected frequency in many cells of the contingency tables will inevitably be less than 5

Re-analyze categorical variables in Table 4 using Fisher's Exact Test instead of the Chi-square test.

Re-analyze continuous variables (particularly LLD) using the non-parametric Mann-Whitney U test to account for non-normality and small subgroup sizes.

Completely restructure Table 4 to include:

Subgroup counts and percentages for all categorical variables.

Subgroup standard deviations (SD) for continuous variables.

Explicit test statistics (e.g., U statistic, t-statistic, df, and 95% Confidence Intervals for differences).

Expand the Limitations section to discuss how the small sample size (N=31) prevented multivariable regression, leaving the significant correlation between LLD and union unadjusted for potential clinical confounders

Reviewer #3: The manuscript addresses an important and challenging pediatric orthopedic condition and presents a relatively large single-center experience with CPT over a 12-year period. However, substantial revision is required before the findings can be adequately interpreted.

INTRODUCTION

• Introduction is too brief and would benefit from further elaboration.

• Expand on the clinical significance and natural history of CPT.

• Provide more background on the challenges of achieving and maintaining union.

• Briefly summarize the main surgical options currently used for CPT.

• Discuss the wide variation in reported union and refracture rates.

• Highlight the limitations or gaps in the existing literature.

• The sentence “The pathology involves poorly perfused invasive hamartomatous fibrous tissue in the surrounding periosteum having increased osteoclasis, low osteogenesis and bone morphogenetic protein” must be revised.

• The inclusion of bone morphogenetic protein in this sentence is unclear and appears incomplete.

• Please clarify the specific role or alteration of BMP in CPT pathology (e.g., reduced expression/activity), rather than simply listing it alongside increased osteoclasis and reduced osteogenesis.

• If the authors are specifically referring to BMP-2, this should be clearly stated and supported by the cited reference.

METHOD

• Consider dividing the Methods into subsections such as Study design and setting; Patient selection; Surgical indications and techniques; Postoperative management and follow-up; Outcome definitions; Ethical approval; and Statistical analysis

• The Methods section requires substantial elaboration to allow reproducibility and proper interpretation of the surgical outcomes.

• The process of patient identification and selection should be described more clearly. Since this is a retrospective study, the authors should explain how eligible CPT cases were identified from the hospital database, how many records were screened, and the reasons for exclusion.

• Please clarify whether all consecutive eligible patients treated during the study period were included, or whether any sampling method was used.

• A clear patient selection flowchart should be provided, showing the number of patients screened, excluded, managed non-operatively, operated on, lost to follow-up, and included in the final union/refracture analysis.

• The indications for surgery are not adequately described. Please specify how the decision for operative treatment was made, including age, fracture status, deformity severity, previous treatment, limb-length discrepancy, or other relevant factors.

• The criteria used to select each surgical technique are unclear. The manuscript describes periosteal grafting, Masquelet technique, Ilizarov fixation, and cross-union, but does not explain why one procedure was chosen over another for a particular patient.

• The surgical protocols need more detail, including extent of pseudoarthrosis resection, fixation method, bone graft source, management of the fibula, use of external fixation/casting, and any adjunctive treatment.

• Please indicate whether procedures were performed by the same surgeon or multiple surgeons, and whether the surgical technique changed over the 12-year study period.

• The postoperative protocol is insufficiently described.

• The authors should clarify the minimum and mean follow-up duration required for assessment of union and refracture.

• The definition of union is provided, but it should be clarified who assessed the radiographs, whether assessment was performed independently, and how disagreement was resolved.

• The ethical approval statement should be incorporated clearly within the Methods section, rather than appearing only later in the manuscript.

Overall, the study has potential clinical value, particularly as an institutional experience from a resource-constrained setting, but major revision of the Introduction and particularly the Methods is necessary to establish the scientific rationale, ensure methodological transparency and reproducibility, and allow appropriate interpretation of the reported outcomes.

**********

what does this mean?). If published, this will include your full peer review and any attached files.

If you choose “no”, your identity will remain anonymous but your review may still be made public.

**Do you want your identity to be public for this peer review?**  For information about this choice, including consent withdrawal, please see our Privacy Policy

Reviewer #1: **Yes:**  Mahmoud Alfatafta

Reviewer #2: **Yes:**  WAFA M. AL-MADHAGI

Reviewer #3: No

**********

[NOTE: If reviewer comments were submitted as an attachment file, they will be attached to this email and accessible via the submission site. Please log into your account, locate the manuscript record, and check for the action link "View Attachments". If this link does not appear, there are no attachment files.]

To ensure your figures meet our technical requirements, please review our figure guidelines: https://journals.plos.org/plosone/s/figures

You may also use PLOS’s free figure tool, NAAS, to help you prepare publication quality figures: https://journals.plos.org/plosone/s/figures#loc-tools-for-figure-preparation.

NAAS will assess whether your figures meet our technical requirements by comparing each figure against our figure specifications.

# Author comment: Author response to Decision Letter 1

**DOI:** 10.1371/journal.pone.0362359.r002

9 Sep 2026

Reviewer 1

Dear reviewer,

We have expanded on the introduction section. We have added literature review as well.

We have revised the methods section. We have tried to make it clearer. The criteria for identification, data collection methodology, follow up assessment and the overall workflow has been defined according to the suggestions made.

This is a retrospective data and we have collected the data regarding the surgery done in our cases. The use of different surgical techniques reflects evolving surgical approaches informed by prior failures and emerging evidence, with the aim of improving patient outcomes at the study center and has been added to the manuscript. However, clear indications for the selection of each technique were not consistently documented in the database, and therefore could not be reported. This has been added to the limitation section.

Due to small sample size we could not perform multivariable analysis. This has now been listed under the limitation section.

We have been more cautious in interpretation of our findings in the discussion and changed the statements accordingly.

We have added the follow up data.

We have done careful proofreading and checked for grammatical errors.

Reviewer 2

Dear reviewer,

We apologize for the typo. We have corrected it.

The contradictions in the table and the abstract has been resolved. The data of the table is the correct one.

Dear reviewer,

we apologize for the typing error. But the file downloaded from the system failed to show such typo. However, we have made sure that it does not occur.

We have rephrased the statement to correct our over statement.

We carried out the analysis again. But the contingency table has all the cells with more than expected counts(>5 per cells). Hence, we continued with our chi square test.

We checked the data for normality and carried out the Mann-Whitney U test which has been explicitly listed in the table 4.

We have reanlayzed the data and presented the result accordingly. We have included the Median and IQR for the non normal continuous data. For the categorical data we have used the pearson’s chi square test.

We have added to the limitation section about the lack of multivariate analysis.

Reviewer 3

Dear reviewer,

• We have expanded on the introduction section. The natural history, presentation and the surgical options have been discussed explicitly now.

• The sentence “The pathology involves poorly perfused invasive hamartomatous fibrous tissue in the surrounding periosteum having increased osteoclasis, low osteogenesis and bone morphogenetic protein” has been revised.

• The statement regarding the BMP had been revised to make it clearer.

We have added substantially to the methods section. Since all the required data could not be located in the database ,appropriately subsections could not be managed. However, ethical statement has been added to the methods section.

• We have elaborated on the methods section to make it clearer to the reader and allow for the reproducibility.

• The patients selection criteria has been listed. The overall flow diagram of the patients has been shown in the figure 1.

• The total sampling method was applied which has been added to the methods section.

• The flowchart is given in the figure 1

• The use of different surgical techniques reflects evolving surgical approaches informed by prior failures and emerging evidence, with the aim of improving patient outcomes at the study center and has been added to the manuscript. However, clear indications for the selection of each technique were not consistently documented in the database, and therefore could not be reported. This has been added to the limitation section.

• The surgical protocols have been elaborated.

• The cases were managed by the same orthopedic team involving multiple surgeons. However the surgical techniques for each procedures followed was the same. This has been explained and added to the manuscript.

• The post operative protocol followed has been listed in the manuscript.

We have added the median follow-up duration.

The bony union was confirmed by two senior authors. The patient’s hospital records were reviewed to confirm the documentation of the clinical union, that was defined as the absence of pain at the fracture site on weight bearing, no tenderness at fracture site on palpation, and ability to bear weight

This has been added to the manuscript

We have moved the ethical statement to the methods section.

Attachment Submitted filename: Response to reviewers 1.pdf

# Peer review: Decision Letter 1

**DOI:** 10.1371/journal.pone.0362359.r003

**Authors:** Xiaoen Wei

2 Oct 2026

Dear Dr. Pokharel,

Thank you for submitting your manuscript to PLOS One. After careful consideration, we feel that it has merit but does not fully meet PLOS One’s publication criteria as it currently stands. Therefore, we invite you to submit a revised version of the manuscript that addresses the points raised during the review process.

Please submit your revised manuscript by Nov 16 2026 11:59PM. If you will need more time than this to complete your revisions, please reply to this message or contact the journal office at plosone@plos.org. When you're ready to submit your revision, log on to https://www.editorialmanager.com/pone/ and select the 'Submissions Needing Revision' folder to locate your manuscript file.

- A letter that responds to each point raised by the academic editor and reviewer(s). You should upload this letter as a separate file labeled 'Response to Reviewers'.
- A marked-up copy of your manuscript that highlights changes made to the original version. You should upload this as a separate file labeled 'Revised Manuscript with Track Changes'.
- An unmarked version of your revised paper without tracked changes. You should upload this as a separate file labeled 'Manuscript'.

If you would like to make changes to your financial disclosure, please include your updated statement in your cover letter. Guidelines for resubmitting your figure files are available below the reviewer comments at the end of this letter.

If applicable, we recommend that you deposit your laboratory protocols in protocols.io to enhance the reproducibility of your results. Protocols.io assigns your protocol its own identifier (DOI) so that it can be cited independently in the future. For instructions see: https://journals.plos.org/plosone/s/submission-guidelines#loc-laboratory-protocols. Additionally, PLOS One offers an option for publishing peer-reviewed Lab Protocol articles, which describe protocols hosted on protocols.io. Read more information on sharing protocols at https://plos.org/protocols?utm_medium=editorial-email&utm_source=authorletters&utm_campaign=protocols.

As the corresponding author, your ORCID iD is verified in the submission system and will appear in the published article. PLOS supports the use of ORCID, and we encourage all coauthors to register for an ORCID iD and use it as well. Please encourage your coauthors to verify their ORCID iD within the submission system before final acceptance, as unverified ORCID iDs will not appear in the published article. *Only*  the individual author can complete the verification step; PLOS staff *cannot*  verify ORCID iDs on behalf of authors.

We look forward to receiving your revised manuscript.

Kind regards,

Xiaoen Wei

Academic Editor

PLOS One

Journal Requirements:

If the reviewer comments include a recommendation to cite specific previously published works, please review and evaluate these publications to determine whether they are relevant and should be cited. There is no requirement to cite these works unless the editor has indicated otherwise.

Please review your reference list to ensure that it is complete and correct. If you have cited papers that have been retracted, please include the rationale for doing so in the manuscript text, or remove these references and replace them with relevant current references. Any changes to the reference list should be mentioned in the rebuttal letter that accompanies your revised manuscript. If you need to cite a retracted article, indicate the article’s retracted status in the References list and also include a citation and full reference for the retraction notice.

[Note: HTML markup is below. Please do not edit.]

Reviewers' comments:

Reviewer's Responses to Questions

**Comments to the Author**

Reviewer #1: All comments have been addressed

Reviewer #2: All comments have been addressed

**********

2. Is the manuscript technically sound, and do the data support the conclusions?

Reviewer #1: Partly

Reviewer #2: Yes

**********

3. Has the statistical analysis been performed appropriately and rigorously?

Reviewer #1: Yes

Reviewer #2: Yes

**********

4. Have the authors made all data underlying the findings in their manuscript fully available?

The PLOS Data policy

Reviewer #1: Yes

Reviewer #2: Yes

**********

5. Is the manuscript presented in an intelligible fashion and written in standard English?

Reviewer #1: Yes

Reviewer #2: Yes

**********

Reviewer #1: Two issues remain that should be addressed before publication.

1. Follow-up duration and classification of non-union: The surgically managed patients had a median follow-up of 21 months (IQR 28), but the reported range is very wide, from 1 to 72 months. A follow-up of only one month would generally be insufficient to establish a definitive surgical outcome, particularly non-union or subsequent refracture. This issue is particularly important because the Discussion states that union has not yet been confirmed in some recently operated cases that remain under follow-up. The authors should clarify how many patients had relatively short follow-up and whether such patients were classified as non-union. If patients with insufficient follow-up were included within the non-union group, the denominator and reported overall union rate should be reconsidered or, at minimum, clearly justified.

2. Interpretation of outcomes between surgical techniques: The authors appropriately acknowledge that clear indications for selecting the different surgical techniques were not consistently documented. This is an important limitation because treatment allocation was therefore neither standardized nor controlled. Consequently, the reported union rates for periosteal grafting, Masquelet technique, and the other procedures should remain descriptive and should not imply comparative effectiveness between techniques. This limitation should remain clear throughout the Results, Discussion and Conclusions.

Reviewer #2: author has addressed all the required comments and also improve the writing as requested from my previous comments

**********

what does this mean?). If published, this will include your full peer review and any attached files.

If you choose “no”, your identity will remain anonymous but your review may still be made public.

**Do you want your identity to be public for this peer review?**  For information about this choice, including consent withdrawal, please see our Privacy Policy

Reviewer #1: **Yes:**  Mahmoud Alfatafta

Reviewer #2: **Yes:**  Wafa M. Al Madhagi

**********

[NOTE: If reviewer comments were submitted as an attachment file, they will be attached to this email and accessible via the submission site. Please log into your account, locate the manuscript record, and check for the action link "View Attachments". If this link does not appear, there are no attachment files.]

To ensure your figures meet our technical requirements, please review our figure guidelines: https://journals.plos.org/plosone/s/figures

You may also use PLOS’s free figure tool, NAAS, to help you prepare publication quality figures: https://journals.plos.org/plosone/s/figures#loc-tools-for-figure-preparation.

NAAS will assess whether your figures meet our technical requirements by comparing each figure against our figure specifications.

# Author comment: Author response to Decision Letter 2

**DOI:** 10.1371/journal.pone.0362359.r004

17 Oct 2026

Dear reviewer, thank you highlighting this important point. We have one case with short follow up. But we could not exclude the patient for analysis, as it would introduce selection bias among the surgically managed cases. However, the effect it being classified as the non union might have slightly lowered the union rates and the refracture rates. This point has been added to the result section and acknowledged in the limitation section as well.

Dear reviewer, thank you for a very valid point. We were very cautious in interpretation of the union rates among different procedures. We tried to avoid the comparison between procedures. However, we have used descriptives in each surgical strategies.

Attachment Submitted filename: Response to reviewers 2.pdf

# Editor report: Decision Letter 2

**DOI:** 10.1371/journal.pone.0362359.r005

**Authors:** Xiaoen Wei

25 Oct 2026

The Surgical Outcome Among Children with Congenital Pseudoarthrosis of Tibia: Our 12 Years of Experience.

PONE-D-26-35562R2

Dear Dr. Pokharel,

We’re pleased to inform you that your manuscript has been judged scientifically suitable for publication and will be formally accepted for publication once it meets all outstanding technical requirements.

Within one week, you’ll receive an e-mail detailing the required amendments. When these have been addressed, you’ll receive a formal acceptance letter and your manuscript will be scheduled for publication.

An invoice will be generated when your article is formally accepted. Please note, if your institution has a publishing partnership with PLOS and your article meets the relevant criteria, all or part of your publication costs will be covered. Please make sure your user information is up-to-date by logging into Editorial Manager at Editorial Manager® and clicking the ‘Update My Information' link at the top of the page. For questions related to billing, please contact billing support.

If your institution or institutions have a press office, please notify them about your upcoming paper to help maximize its impact. If they’ll be preparing press materials, please inform our press team as soon as possible -- no later than 48 hours after receiving the formal acceptance. Your manuscript will remain under strict press embargo until 2 pm Eastern Time on the date of publication. For more information, please contact onepress@plos.org.

Kind regards,

Xiaoen Wei

Academic Editor

PLOS One

Additional Editor Comments (optional):

Reviewers' comments:

# Editor report: Acceptance letter

**DOI:** 10.1371/journal.pone.0362359.r006

**Authors:** Xiaoen Wei

PONE-D-26-35562R2

PLOS One

Dear Dr. Pokharel,

I'm pleased to inform you that your manuscript has been deemed suitable for publication in PLOS One. Congratulations! Your manuscript is now being handed over to our production team.

At this stage, our production department will prepare your paper for publication. This includes ensuring the following:

* All references, tables, and figures are properly cited

* All relevant supporting information is included in the manuscript submission,

* There are no issues that prevent the paper from being properly typeset

You will receive further instructions from the production team, including instructions on how to review your proof when it is ready. Please keep in mind that we are working through a large volume of accepted articles, so please give us a few days to review your paper and let you know the next and final steps.

Lastly, if your institution or institutions have a press office, please let them know about your upcoming paper now to help maximize its impact. If they'll be preparing press materials, please inform our press team within the next 48 hours. Your manuscript will remain under strict press embargo until 2 pm Eastern Time on the date of publication. For more information, please contact onepress@plos.org.

You will receive an invoice from PLOS for your publication fee after your manuscript has reached the completed accept phase. If you receive an email requesting payment before acceptance or for any other service, this may be a phishing scheme. Learn how to identify phishing emails and protect your accounts at https://explore.plos.org/phishing.

If we can help with anything else, please email us at customercare@plos.org.

Thank you for submitting your work to PLOS One and supporting open access.

Kind regards,

PLOS One Editorial Office Staff

on behalf of

Dr. Xiaoen Wei

Academic Editor

PLOS One
